# Reactivity to allergenic food contaminants: A study on products on the market

**DOI:** 10.1002/clt2.12301

**Published:** 2023-09-22

**Authors:** Alessandro Fiocchi, Linda Monaci, Elisabetta De Angelis, Veronica Calandrelli, Lamia Dahdah, Rocco Valluzzi, Sara Urbani, Carmen Mazzuca, Stefania Arasi, Arianna Cafarotti, Carla Riccardi, Maria Cristina Artesani, Lorenza Putignani, Valentina Pecora, Valeria Marzano, Vincenzo Fierro

**Affiliations:** ^1^ Allergy Dpt Bambino Gesù Children's Hospital IRCCS Rome Italy; ^2^ Institute of Sciences of Food Production CNR‐ISPA Bari Italy; ^3^ Unit of Microbiology and Diagnostic Immunology Bambino Gesù Children's Hospital IRCCS Rome Italy; ^4^ Unit of Human Microbiome Department of Diagnostics and Laboratory Medicine Bambino Gesù Children's Hospital IRCCS Rome Italy

**Keywords:** allergen toxicity, food allergy, hazelnut allergy, milk allergy, precautionary allergen labelling

## Abstract

**Background:**

The frequency and severity of reactions in food‐allergic consumers exposed to unintentional food allergen contamination during production is unknown. To warn allergic consumers, it has been suggested for pre‐packaged foods to be precautionary labelled when the food allergen contamination may exceed the amount to which 1%–5% of the population could react (ED01–ED05). ED01 for hazelnut and milk have been estimated at 0.1 and 0.2 mg, respectively, by the Voluntary Incidental Trace Allergen Labelling (VITAL) initiative. The respective reference doses recommended by the FAO/WHO Codex consultation are 3 and 2 mg. We evaluated the reactivity to potential traces of milk and hazelnut allergens in allergen‐free pre‐packaged products by children affected by severe allergies to milk and hazelnuts.

**Methods:**

Oral Food Challenges with commercially available hazelnut‐free wafer biscuits and milk‐free chocolate pralines were administered to patients with severe food allergies to hazelnut and cow's milk, respectively. Contamination levels of milk or hazelnut allergens were measured using chromatographic separation interfaced with triple quadrupole mass spectrometry.

**Results:**

No hazelnut allergic patient showed allergic reactions to exposure to biscuits, nor any milk allergic patient displayed allergic reactions to the dark chocolate praline. While no hazelnut trace was detected in biscuits, the praline was found to be contaminated by milk at concentrations ranging between 8 and 35 mg total protein/kg food. In our dose model, these amounts exceeded 1.5–10 times the VITAL ED01 and reached the threshold suggested by the FAO/WHO Codex consultation.

**Conclusions:**

Upon the consumption of food products available on the market, many patients with severe food allergies tolerate significantly higher doses of allergen than reference doses indicated in the VITAL system used for precautionary allergen labelling. These doses support the safety of the FAO/WHO recommended reference doses.

## 1 INTRODUCTION

Food trace reactions have been reported in 18% of children^1^ and in 32% of adults with food allergy.^2^ In such patients, tree nuts, cow's milk, and peanuts are the most common suspected contaminating allergens. In a significant number of countries, if such foods are contained as an ingredient in a pre‐packaged product they are required to be reported on the label as allergens by law.^3^, ^4^ If the same allergen is not an ingredient of the pre‐packaged product, but may contaminate it, many manufacturers choose to communicate potential allergen presence using Precautionary Allergen Labelling (PAL). Precautionary Allergen Labelling is an unregulated, voluntary practice aimed to protect consumers suffering from IgE‐mediated food allergy from unexpected reactions that could also be potentially dangerous.

As PAL poses several problems to allergic consumers,^5^, ^6^, ^7^, ^8^ the international authorities together with the scientific community are attempting to discipline this matter. In 2019, the FAO/WHO Codex Committee on Food Labelling asked for scientific advice aimed to develop guidance on the use of PAL. The commission set up in response to this appeal proposed risk management and mitigation strategies. The Codex Expert consultation indicated as the most suitable strategy to establish health‐based guidance values for PAL the use of food allergen thresholds based on a dose/probabilistic hazard assessment.^9^ This choice substantially adopted the approach proposed by the Allergen Bureau's Voluntary Incidental Trace Allergen Labelling (VITAL) Programme,^10^ reducing the number of priority allergens. Elaborating on the concept of a minimum amount able to determine allergic reactions, VITAL defined the ‘eliciting doses’ (EDs) for specific food allergens, indicating the dose (mg) predicted to provoke reactions in a defined proportion of the allergic population.^11^, ^12^, ^13^ The VITAL EDs were derived from the dose distribution of individual minimum EDs, based on the results of the Oral Food Challenges (OFCs) performed for diagnostic purposes and published in the scientific literature.^14^ The Codex expert committee proposed thresholds based on the published ED05 values (the dose predicted to provoke reactions in 5% of the at‐risk allergic population)^11^ as the most appropriate reference doses to guarantee the transparency of information to the consumer and safeguard his safety.^15^

A decisional process for PAL based on EDs for specific food allergens makes sense, as (I) the probability to have a reaction depends on the EDs,^16^ (II) the EDs depend on the type of food,^17^ (III) OIT (Oral Immunotherapy) studies indicate that increasing the ED reduces the risk of an allergic reaction from accidental exposure,^18^ and (IV) the use of anti‐IgE reduces the likelihood and the severity of the reactions by increasing the threshold doses for different foods.^19^ However, up to now, translating these indications into practical applications has proven difficult.^14^ Among other difficulties, VITAL has not been adopted on a large scale due to the difficulties in verifying it with the current analytic capabilities.^20^ On the other hand, the suspicion hovers that the VITAL thresholds may be overconservative, because the source data of the project refer to populations of patients allergic to foods *tout court*, and not to populations of severe food allergic.^21^ It has been observed that no fatal reactions have ever occurred after exposure to allergen concentration of less than 5 mg of protein for any allergen, although allergic—but not life‐threatening—reactions can occur at such levels.^22^ From this observation, the European GA2LEN network proposed a potential conservative threshold/risk management cut‐off of five parts per million (ppm) or 0.5 mg referred to 100 g of food size. Such proposed threshold far exceeds the VITAL thresholds for many of the allergenic foods considered, and has been criticized as inaccurate.^23^

Under this uncertainty frame, studies are lacking aiming at defining the frequency and severity of reactions in severe food‐allergic patients exposed to doses below the FAO/WHO reference dose, the ED01 threshold set by VITAL, the GA2LEN threshold, or the recent reference dose of 0.3 mg (ED01) recommended for total milk proteins for risk assessment and management purposes.^13^

The present study is an attempt to contribute to fill this lack of knowledge. In a ‘real life’ setting, we aimed to verify the reactivity to potential traces of milk and hazelnut allergens in milk and hazelnut‐free products in a group of children affected by severe allergies to milk and hazelnuts.

## 2 METHODS

### 2.1 Study products and clinical outcomes

The study was designed to evaluate whether patients with severe food allergy might experience clinical reactions to two commercially available pre‐packaged foods: hazelnut‐free cocoa and milk wafer biscuits with milk and cocoa fillings and a milk‐free chocolate confectionery praline. Such cookies are labelled as “may contain hazelnut traces” and “may contain milk traces”, respectively. Aiming at evaluating any relationship existing between the potential doses of food allergens traces contained in the studied products and expected reactions that are likely to occur in case of cross‐contamination by allergens, we carried out in parallel a clinical and chemical evaluation. Mass spectrometric analysis was carried out to quantify potential traces of hazelnut and milk detected in the biscuit and in the dark chocolate praline, respectively, and the potential reactivity of the contaminated products was evaluated by the OFC test.

### 2.2 Population

We admitted to the study milk/hazelnut protein allergic children aged between 2 and 18 years with history of anaphylaxis, defined as a systemic food hypersensitivity reaction, rapid in onset and potentially fatal at any time in the past.^24^ Participants must have received a prescription of epinephrine, whether they had used it or not. They had to be sensitized to hazelnut or milk by both skin test (cut‐off greater than 3 mm wheal^25^) and specific IgE dosage for milk or hazelnut (ImmunoCAP Thermo Fisher, Uppsala, Sweden, cut‐off >0.35 kUI/L). Patients also had to show IgE positivity for at least one of the major molecular allergens: for CMA, Bos d 4, Bos d 5, and/or Bos d 8; for hazelnut allergy sufferers, Cor a 8, Cor a 9, and/or Cor a 14 (ImmunoCAP, cut‐off >0.35 kUI/L). None of them was allowed to consume products containing traces of the respective food allergens.

The patients underwent confirmatory seven steps of OFC using pasteurized low‐fat milk or raw chopped hazelnut. Specifically, for milk, we set the lowest dose to 3.43 mg of protein and the total amount of protein to 4955.8 mg, corresponding to 0.1 and 144.4 mL, respectively. For hazelnut, we set the lowest dose to 13.8 mg and the total amount of protein at 4222.8 mg of proteins (about 0.1 and 30.6 g, corresponding to 20 hazelnuts) (Table 1). We derived the protein content of the foods from the U.S. Department of Agriculture.^26^ The doses were administered 20 min apart until objective symptoms appeared.^27^, ^28^ We stopped the administration when objective symptoms appeared, according to the scheme shown in Table SI. The reactive symptoms classified patients into five groups, from subjective (nausea, abdominal pain, pruritus, oral allergy syndrome) to systemic reactions (Table SII).^29^

**TABLE 1:** Equivalent amounts of milk and hazelnut used in diagnostic Oral Food Challenges (OFCs).

Step dose	Raw chopped hazelnut	Hazelnut protein	Pasteurized cow's milk	Cow's milk protein
1	150 mg	20.7 mg	0.1 mL	3.43 mg
2	390 mg	51.06 mg	0.3 mL	10.29 mg
3	500 mg	69 mg	1 mL	34.32 mg
4	1000 mg	138 mg	3 mL	102.96 mg
5	2000 mg	276 mg	10 mL	343.2 mg
6	4000 mg	552 mg	30 mL	1029.6 mg
7	8000 mg	1104 mg	100 mL	3432 mg
**Cumulative dose**	**16.040 g**	**2192.13 mg**	**144.4 ml**	**4955.80 mg**

In order to include clinically relevant patients for our query, admission to the next phase of the protocol was limited to patients who reacted to the first, second, or third dose of OFC, that is, those allergic to up to 1 gm hazelnut or up to 1.4 mL of milk.

Children, or their parents or legal guardian, were required to sign a statement of informed consent that met the privacy requirements including those of the GDPR. The local ethics committee gave ethical approval to the protocol (Resolution #851, July 30th, 2018).

### 2.3 Procedures

Two groups of participants were evaluated, with (a) hazelnut allergy or (b) CMA. Group (a) was challenged with different batches of wafer biscuits with milk and chocolate filling, group (b) was exposed to different batches of dark chocolate pralines.

In group (a), we performed a SPT (Skin Prick Test) with fresh hazelnut, baked products containing hazelnut and the filled biscuit.^24^ After that, OFCs were administered in seven increasing doses. The first six doses were the same for each age group, the seventh varied according to patient's age. Each dose was administered every 20 min for a duration of 140 min (Table 2). Patients were monitored for 2 h after the administration of the last dose.

**TABLE 2:** Oral Food Challenge (OFC) doses with study products.

Step dose	Group (a)—filled biscuits	Group (b)—dark chocolate pralines
1	0.4 gr	0.56 gr
2	0.8 gr	1.125 gr
3	1.6 gr	2.25 gr
4	3.2 gr	4.5 gr
5	6.4 gr	9 gr
6	12.8 gr	18.0 gr for subjects <5 yo or 27.0 gr for subjects 6–9 yo or 36.0 gr for subjects 10–13 or 45.0 gr for subjects 14–18 yo
7	25.6 gr for subjects <5 yo or 35.6 gr for subjects 6–9 yo or 45.6 gr for subjects 10–13 or 51.2 gr for subjects 14–18 yo	

For group (b) patients, the SPTs with the following products were performed: commercially available cow's milk, alpha‐lactalbumin, betalactoglobulin, casein (Lofarma, Milano, Italy), fresh pasteurized cow's milk, baked products containing milk, and shells of dark chocolate pralines. In this group, OFCs were performed on four portions of chocolate for a total weight of 9 gr of product for each chocolate; OFC was refracted in 6 doses, administered every 20 min, for a total test duration of 100 min (Table 2). Similar to the biscuit protocol, the last dose (i.e. the sixth) changed according to patient's age.

In both groups, the amount of the last dose was commensurate with the age of the children (Table 2). The patients in group (a) assumed a total number of biscuits oscillating between 50.8 gms under 5 years and 76.4 gms over 14 years. Those in group (b) assumed a quantity fluctuating between 35.4 gms for children under 5 years and 62.4 gms for children above 14.

### 2.4 Analytical protocol for the determination of milk allergens in dark chocolate pralines

The analysis for the determination of contamination levels of milk allergens used a method based on chromatographic separation interfaced with triple quadrupole mass spectrometry (LC‐MS/MS).

#### 2.4.1 Dark chocolate pralines milling

For the extraction and analysis of milk allergens in dark chocolate‐based samples, the analytical method based on LC‐MS/MS detection developed within the ThRAll project^30^, ^31^ and validated in house was followed. Briefly, chocolate products were grinded using a laboratory blender and the final mixture was filtered through a 1 mm cut‐off sieve. The obtained powder was stored in the fridge until its use.

#### 2.4.2 Protein extraction, digestion and peptides marker selection

For protein extraction, 2 g of chocolate was extracted with 20 mL of Tris‐HCl 200 mM pH 9.2 and 5M urea and subjected to purification as detailed elsewhere.^32^ An aliquot was then digested with trypsin and the resulting peptide pool purified on SPE StrataX columns (1cc, 30 mg, #8B‐S100‐TAK, Phenomenex Torrance, California, USA) before the final injection into the HPLC‐MS apparatus. The identification of the milk allergenic ingredient in the different samples was achieved by the detection of two main peptides: FFVAPFPEVFGK peptide (FFV, m/z 692.9) belonging to the alpha‐S1 casein protein and NAVPITPTLNR peptide (NAV, m/z 598.3) released from alpha‐S2 casein tryptic digestion, along with the respective transitions monitored in the Multiple Reaction Monitoring (MRM) analyses, namely 920.5/991.5/676.4/823.4 for FFV and 911.5/285.2/701.4/600.3 for NAV. In tab S III, any relevant information about milk peptide markers was reported. Samples were injected in duplicate into the LC/MS system.

#### 2.4.3 Calibration line

For the quantification of the milk ingredient in the dark chocolate samples, a calibration line was constructed by adding calculated amounts of the synthetic peptide FFVAPFPEVFGK (m/z 692.9) to the digest of a milk‐free chocolate sample after purification on SPE columns.

A four specific calibration line was constructed by relating the peak area detected for each transition to the respective concentration expressed in fmol/μL. The quantification of the milk in the individual samples was carried out using the equation of the transition line showing the best sensitivity, that is, the transition 692.9/920.4 by interpolation of the peak areas detected for the transition 920.4 in the different samples.

In order to quantify the milk protein concentration in the analysed product, the FFV peptide concentration (fmol/μL) was converted into micrograms of milk protein by the previously reported correction and conversion factors.^32^

Twenty‐nine samples marked as “Choco_B_01” to “Choco_B_29” were analysed in replicate.

### 2.5 Analytical protocol for the determination of hazelnut allergens in filled biscuits

Snack samples marked as “Biscuits_01” to “Biscuits_30” were processed and analysed by the HPLC/QqQ‐MS according to the method currently described with the aim to detect any trace of hazelnut proteins.

#### 2.5.1 Filled biscuits milling

The products were firstly grinded by using a laboratory blender, in particular, three iterative cycles of pulsed blending at decreasing speed were applied in order to minimize the sample heating (pulses: 5sON/5sOFF + 5sON/5sOFF, speed 16,000 rpm I cycle, 14,000 rpm II cycle, 13,000 rpm III cycle). The obtained flour was sieved through a 2 mm mesh and then stored at −20°C until its use. The procedure was equally applied to all snack samples. Independent blades and containers were used for each sample to avoid cross‐contamination.

#### 2.5.2 Preparation of isotopically labelled synthetic peptides standard solution

An explorative study tailored to confirm the presence/absence of hazelnut allergenic ingredient before quantification was accomplished by using isotopically labelled synthetic peptides (heavy peptides) purchased from Thermo Fisher Scientific (Bremen, Germany). Specifically, isotopic labelling was produced on the terminal Lysine (K) or Arginine (R) of the amino acid sequence to obtain a shift mass with respect to the unlabelled peptides. Heavy peptides (AQUA Basic) were singly provided in lyophilized form to be then re‐suspended in 5% (v/v) acetonitrile/water to get the final concentration of 6250 fmol/μL. Peptide solutions were then divided into different aliquots and stored at −20°C until use. Heavy peptide amino acidic sequences were chosen on the basis of the marker peptides identified within the ThRAll project^30^, ^31^ for tracing hazelnut in food‐processed samples.

#### 2.5.3 Protein extraction, in solution digestion and peptide mixture purification

After grinding, samples were submitted for protein extraction/purification. Specifically, protein extraction was performed as already reported in our previous paper.^33^ Before purification, an aliquot of isotopically labelled synthetic peptides was added to the peptide mixture in order to obtain the final concentration of 25 fmol/μL.

Thirty samples from 12 different lots, marked as “Biscuits_01” to “Biscuits_30”, were finally injected in replicate in LC‐MS/MS equipment.

#### 2.5.4 Peptides for the identification of hazelnut proteins

Table SIV reports the hazelnut peptides chosen as markers for tracing this allergen in the biscuit samples. Specifically, two main peptides were selected for identifying hazelnut in the samples, namely: ADIYTEQVGR peptide (ADI, m/z 576.3) and ALPDDVLANAFQISR peptide (ALP, m/z 815.4) both belonging to the 11S Seed Storage Globulin. The respective transitions monitored in the MRM analyses were 852.4/689.4/588.3/693.3 for ADI and 906.5/835.4/1019.6/1445.7 for ALP. In tab S IV, any relevant information about hazelnut peptide markers is reported. Samples were injected in duplicate into the LC/MS system.

### 2.6 LC‐MS analysis for milk and hazelnut allergen detection

For LC–MS analyses, a system consisting of an LX50 UHPLC pump provided with an autosampler and an ESI interface connected to a QSight 220 triple mass spectrometer (PerkinElmer, Inc., Waltham, MA USA) was used. Peptide separation was accomplished on a Perkin Elmer Brownlee SPP ES‐C18 (2.1 × 150 mm; 2.7 μm; 160 Å) (PerkinElmer Inc., Waltham, MA, USA) at a flow rate of 300 μL/min, using a binary gradient composed by H_2_O + 0.1% formic acid (solvent A) and Acetonitrile + 0.1% formic acid (solvent B). The gradient elution programme was 0–3 isocratic steps at 10% B, then a linear increase from 10% to 35% B in 25 min; step change to 90% B then isocratic for 15 min; step change to 10% B then isocratic for 17 min for column conditioning. For each sample 18 μL was loaded. Mass spectrometer analysis was performed in duplicates in MRM by setting the resolution to “Unit” option (0.7 ± 0.1 amu) for both Q1 and Q3 events. MS conditions were set as reported elsewhere.^33^ Instrumental control and data processing were obtained using the Simplicity ‐ 3Q v. 1.6.5 (PerkinElmer Inc., Waltham, MA, USA) commercial software.

## 3 RESULTS

### 3.1 Clinical data

Between September 1st, 2018, and October 31st, 2022, 207 patients underwent milk and 111 hazelnut OFCs. Six of these procedures, or 1.88%, were DBPCFC. Thirty‐one patients were reported with possible allergic reactions to both milk and hazelnut.

Sixty‐seven (32.4%) of the patients who completed the OFC with milk, and sixty‐five (58.6%) of those with suspected hazelnut allergy gave a positive result (Table 3). The proportion of positive tests was significantly higher for hazelnut (chi‐square = 8.0511, *p* = 0.004). Forty‐two (31.8%) of the positive patients had been reported with a severe systemic reaction at clinical history, according to the established clinical criteria,^25^ without difference among groups (a) and (b).

**TABLE 3:** Demographic characteristics of the 59 patients included in the study.

	Milk	Hazelnut	Total
Age (years), mean (min, max)	9.02 (2.21, 17.85)	8.19 (2.47, 17.43)	8.76 (2.21, 17.85)
Male, *n* (%)	20 (69)	18 (60)	38 (63.3)
Respiratory allergy, *n* (%)	18 (62)	24 (80)	42 (71.2)
Diagnostic challenges performed, n	29	30	59
Open, *n* (%)	143 (97.9)	141 (99.3)	284 (98.6)
DBPCFC, *n* (%)	1 (3.4)	0	1 (1.7)
First dose responders, *n* (%)	1 (3.4)	1 (3.3)	2 (3.4)
Second dose responders, *n* (%)	7 (24.1)	13 (43.3)	20 (33.9)
Third dose responders, *n* (%)	21 (72.4)	16 (53.3)	37 (62.7)

Sixty patients, 38 males and 22 females (mean age 8.76 years, range 2.21–17.85), satisfying the inclusion criteria and reacting to low doses of cow's milk (30) or hazelnut (30), agreed to participate in the study (Table 3). Fifteen of them (20%; 10 in group (a) and 5 in group (b)) reported reactions to suspected allergen(s) not mentioned as ingredient or warning on the label at their clinical history. Only one patient from each of the two groups developed objective symptoms on the first diagnostic OFC dose. Twenty‐eight of them developed at OFC urticarial eruptions and were classified in Group 2, 22 developed Group 3 reactions, and 10 patients developed Group 4 systemic reactions (five with urticaria and dyspnoea, two with urticaria and mild hypotension, one with emesis and dyspnoea, one with emesis with dyspnoea, and one with urticaria dyspnoea and emesis). Eight out of 10 of Group 4 reactions occurred with hazelnut ingestion.

As a girl allergic to milk turned 18 before she could undergo the experimental OFCs and had to be excluded from the evaluation, we report here the data of 59 patients.

In group (a), while all the SPTs with hazelnut were positive (wheal diameter 5–28 mm), the SPTs with the filled biscuits returned negative. In group (b), the SPTs returned positive with the following proportions: alpha‐lactalbumin 41.4% (12/29); betalactoglobulin, 48.3% (14/29); casein, 93.1% (27/29); dark chocolate pralines, 3.4% (1/29). In this group, the range of wheal diameters was between 3 and 25 mm.

At specific IgE determination, patients in group (a) were found to be positive for hazelnut molecular allergens in the following proportions: Cor a 8, 50% (15/30); Cor a 9, 73% (22/30); Cor a 14, 66.6% (20/30). Those with CMA (group b) were positive for Bos d 4 in 44.8% of cases (13/29), Bos d 5 in 55.2% of cases (16/29), and Bos d 8 in 96.5% of cases (28/29).

In study product OFCs, the possible food allergen contamination did not show allergic reactions to the exposure to the biscuits, nor has any milk allergic patient shown allergic reactions to the dark chocolate. Thus, the possible food allergen contamination in the tested products, if any, did not reach clinically significant levels.

### 3.2 Determination of the presence of hazelnut proteins in the filled biscuits

**FIGURE 1:**
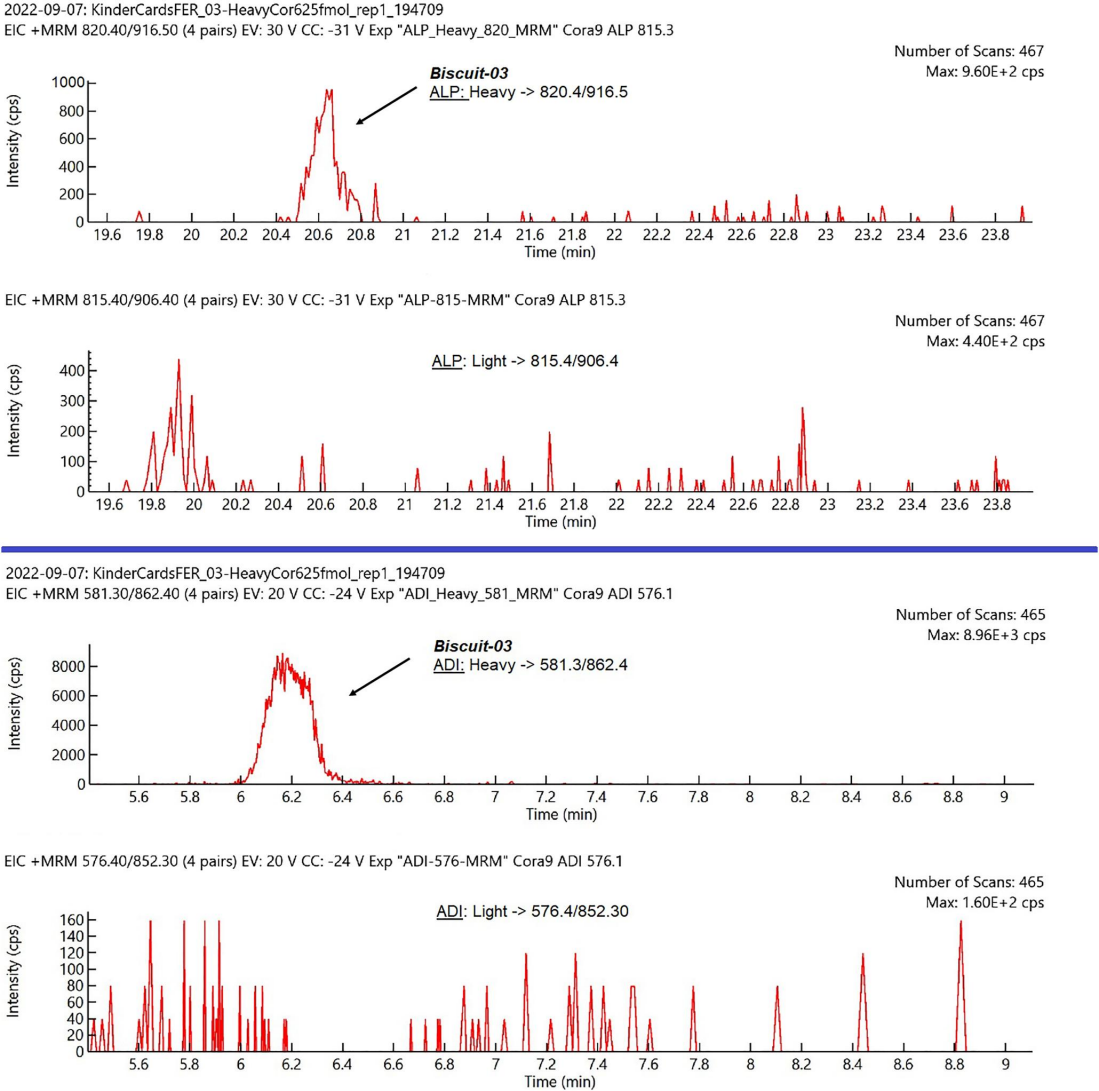
Comparison between Multiple Reaction Monitoring (MRM) signals produced by analysing the Biscuits‐03 sample and monitoring the transition 815.4/906.4 m/z for ALP (plot A) and 576.4/852.3 for ADI (plot B) peptides in labelled (heavy) and unlabelled (light) forms.

The MRM analysis of both marker peptides revealed that no hazelnut trace was present in any sample analysed (Biscuits 1–30). Comparing the chromatogram plots produced by LC‐MS/MS analysis of each sample by monitoring each single transition derived from ADI and ALP precursors in unlabelled and labelled forms, no peak areas corresponding to hazelnut peptides were revealed or, in any case, they were found below the signal produced by the presumable minimum detectable concentration. An example of the chromatogram peaks produced by LC‐MS/MS analysis of the Biscuits‐03 sample referred to a selected transition of ALP and ADI unlabelled/labelled peptides is reported in Figure 1, plots A and B, respectively.

### 3.3 Determination of the presence of milk proteins in dark chocolate pralines

Table 4 shows the concentration values of milk proteins estimated on the quantitative analysis obtained by LC‐MS, after the application of the conversion factors and correction to the dark chocolate samples analysed. In this case, the samples (*n* = 29) were actually found to be contaminated by small quantities of milk, ranging between 8 and 35 mg total milk protein/kg food. In light of this finding, our patients assumed during OFC doses of milk proteins ranging from 0.20 to 2.18 mg, as indicated in Table 5.

**TABLE 4:** Milk protein concentration assessed in dark chocolate praline samples by LC‐MS after application of appropriate conversion factors.

Sample	Marker concentration (fmol peptide/μl extract)	Milk protein concentration^a^ corrected by the conversion and pre‐concentration factor (mg total milk protein/kg food)
Choco_B01_030322	273	21
Choco_B02_030322	293	23
Choco_B03_080322	365	28
Choco_B04_080322	320	24
Choco_B05_080322	327	25
Choco_B06_080322	303	23
Choco_B07_080322	331	25
Choco_B08_030322	451	35
Choco_B09_030322	390	30
Choco_B10_090322	286	22
Choco_B11_090322	168	13
Choco_B12_090322	199	15
Choco_B13_090322	222	17
Choco_B14_040322	230	18
Choco_B15_040322	142	11
Choco_B16_090322	257	20
Choco_B17_150322	177	13
Choco_B18_150322	206	16
Choco_B19_150322	202	16
Choco_B20_150322	231	18
Choco_B21_040322	107	8
Choco_B22_150322	179	14
Choco_B23_150322	149	11
Choco_B24_150322	123	9
Choco_B25_150322	161	12
Choco_B26_150322	162	12
Choco_B27_150322	140	11
Choco_B28_150322	160	12
Choco_B29_150322	160	12

^a^ Milk concentrations were rounded up.

**TABLE 5:** Amount of milk protein by group (b) (dark chocolate pralines) consumed during experimental Oral Food Challenge (OFC) in different age groups.

Patient age, years	Chocolate dose	Milk protein min, μg	Milk protein max, μg
<5	35.2 gr	281.6	1232
6–9	44.2 gr	353.6	1547
10–13	53.2 gr	425.6	1862
14–18	62.2 gr	497.6	2177

## 4 DISCUSSION

The aim of this study was to verify whether, in a population of individuals severely allergic to milk or hazelnut, exposure to foods potentially contaminated by the allergen could cause significant reactions. Unlike all other studies evaluating low‐dose reactivity, its design was not a retrospective evaluation of the thresholds of foods in the diagnostic OFCs, but a prospective exposure to potentially contaminated foods. As it was conceived without a predetermined contamination of the tested foods, this was a real‐life study.

The tested products proved to be safe on this highly selected population. As none of the 59 children had reactions, the potential contaminating allergens did not reach clinically relevant levels.

For group (a), the absence of a measurable hazelnut contamination does not allow us to draw different conclusions from those already reached for milk and egg.^26^ In fact, for hazelnut, the absence of traces deemed quantifiable by mass spectrometry reassures about the safety of exposure to these filled biscuits.

Conversely, the chocolate administered in OFCs to group (b) exhibited actual milk contamination, ranging between 8 and 35 mg total protein/kg food. These concentrations exceed by 1.5–6 times the threshold of 5 mg total milk protein/kg food proposed by GA2LEN for the voluntary declaration of traces of food allergens in processed foods.^21^ The NOAEL for our patients was found at amounts up to 10 times the VITAL ED01^11^ and reached the FAO/WHO threshold. In this study, the exposure to such amount of milk traces in chocolate pralines was not detrimental to the health of children with severe CMA. As chocolates and bonbons, and especially milk in dark chocolate products, are among the most frequently reported causes of reactions to trace foods,^2^ our finding is reassuring about the specific safety of the product. More in general, it raises questions about the real opportunity to apply precautionary labelling to similar pre‐packaged foods.

In fact, the precautionary thresholds calculated from the VITAL collaboration are based on retrospective studies of tests performed for diagnostic purposes. Such OFCs are not specifically designed to evaluate thresholds; their results may vary with several factors, including the challenge technique, the age of patients and the case‐mix of individual centres. It is reasonable to expect that centres serving patients with less severe food allergies may have a higher reactivity threshold, whereas third‐level centres could have lower thresholds. Nevertheless, in a third‐level centre such as the Bambino Gesù Paediatric Hospital, we recently published milk thresholds of 0.3 mg of total protein.^17^ In a similar setting, the discrete ED01 for milk was found at 1.1 mg.^33^ Such values exceed the threshold for milk by 1.5 and 5.5 times, respectively.

Thus, it is not impossible to hypothesize that the VITAL thresholds are over‐conservative. This hypothesis is supported by the conclusions of the FAO/WHO expert consultation that considered ED05‐values the appropriate basis for establishing health‐based guidance values (reference doses). The results of our study support the safety of the ED05‐based reference dose for milk as recommended by the FAO/WHO expert consultation.^9^

European, U.S., Australian, and Canadian regulations require food business operators to implement controls to manage allergens and ensure they are properly declared, but the use of PAL is voluntary. In none of these countries have the regulatory authorities suggested quantitative thresholds below which a PAL is not necessary. This may encourage the food producers to be overcautious, as they are responsible for any accidents caused by contamination of undeclared food allergens. Should our results be confirmed, a re‐thinking of this legislation could be possible. However, to consolidate these data, it will be necessary to prospectively evaluate the reaction of populations of severely allergic patients to foods contaminated with tiny, increasing doses of food allergens. This method, widely used to evaluate the thresholds of disparate biological phenomena,^34^, ^35^, ^36^ could identify ‘no‐observed‐adverse‐effect‐concentrations (NOAECs)’ of food allergens and might provide additional proof of the validity of ED‐distributions^11^, ^12^ and the safety of the ED05‐based reference doses recommended by the FAO/WHO expert consultation.^9^

The results of this study allow ancillary considerations.

First, we used an unprecedented reaction severity rating, including a history of anaphylactic reactions, the prescription of epinephrine, confirmed sensitization, and sensitization to allergens considered highly hazardous.^37^ To the best of our knowledge, such stringent inclusion criteria have not been used in previous studies. We believe that this type of patient can be further exploited in future studies to investigate reactivity to allergens contaminating food. The recently published definition of Severe Food Allergy^38^ will help identify patients who require an absolute exclusion of foods containing traces.

Second, a significant proportion of patients undergoing OFCs in paediatric allergy service manifests low‐dose reactions. In our series, this proportion is different for cow's milk (32%) and hazelnut (58%). While observation coincides with the concept that nut reactions can occur at extremely low thresholds,^16^ the severity of the reactions was not different in the two groups.

Third, the percentage of patients allergic to Cor a 14 and Cor a 9 is a relevant in group (a), while sensitization to Bos d 8 is almost universal in group (b). Many studies accomplished on hazelnut allergy indicated the association between sensitization to the structural proteins Cor a 9 (legumin) and Cor a 14 (2S‐albumin) and the occurrence of anaphylactic reactions.^38^, ^39^, ^40^, ^41^ Likewise, Bos d 8 (casein) has been since a long time indicated as a key molecular allergen for CMA.^42^, ^43^

The major limitation of this study was its design as a real‐life study. This choice was made deliberately in an attempt to make data available to the scientific community resulting from the ordinary exposure to which patients with severe food allergies are subject. However, given the absolute absence of hazelnut contamination in the wafers, in one of its arms the results could be compared with the current reactivity thresholds. Another possible limitation is that our sample ‐ although made up of patients who reacted to low doses ‐ cannot fail to include patients who reacted to trigger doses of up to 70 mg of protein. While this value is certainly higher than the trace values, this is the only way to characterize a low‐dose reactive patient population. This threshold does not exclude that our patients were reactive to traces, and they had reported a frequency of reactions to foods not present as ingredients comparable to that of other reported populations.^1^, ^2^ Perhaps the major limitation, however, is our limited size of population of food‐allergic children screened, and the fact that this is made of milk‐allergic children only. This does not allow the study to be elevated to a universal standard.

In conclusion, our data may yield the hypothesis that reaction thresholds to allergens contaminating food products, at least in the case of milk, may be higher than estimated so far. To be confirmed, they will need to be replicated on severely allergic individuals exposed to minute, known, and increasing amounts of allergenic contaminants aiming to identify NOAELs. Only by following this approach would it be possible to establish a real scale of danger referred to the amount of the contaminating allergen in a specific food. In a context where the practice of PAL is widely extended, reducing the possibilities of food choices of allergic consumers, this approach could lead research to go against the tide.

## AUTHOR CONTRIBUTIONS

**Alessandro Fiocchi**: Conceptualization (lead); Data curation (supporting); Formal analysis (supporting); Funding acquisition (lead); Investigation (supporting); Methodology (lead); Project administration (lead); Resources (supporting); Supervision (lead); Validation (equal); Writing – original draft (lead). **Linda Monaci**: Data curation (lead); Investigation (equal); Writing – original draft (equal). **Veronica Calandrelli**: Data curation (equal); Investigation (equal). **Elisabetta De Angelis**: Investigation (equal). **Lamia Dahdah**: Investigation (equal); Writing – review & editing (equal). **Rocco Valluzzi**: Data curation (equal); Formal analysis (equal); Methodology (equal); Writing – review & editing (equal). **Sara Urbani**: Investigation (equal). **Carmen Mazzuca**: Data curation (equal); Investigation (equal). **Stefania Arasi**: Data curation (equal); Visualization (equal); Writing – review & editing (equal). **Arianna Cafarotti**: Investigation (equal). **Carla Riccardi**: Investigation (equal). **Maria Cristina Artesani**: Investigation (equal). **Lorenza Putignani**: Investigation (equal). **Valentina Pecora**: Writing – review & editing (equal). **Valeria Marzano**: Investigation (equal). **Vincenzo Fierro**: Conceptualization (equal); Investigation (equal); Project administration (equal); Writing – review & editing (equal).

## CONFLICT OF INTEREST STATEMENT

Alessandro Fiocchi: Danone SA, Aimmune, Hipp GmbH, Abbott SA, Novartis, Ferrero, Sanofi. Linda Monaci: Barilla. Veronica Calandrelli: none. Elisabetta De Angelis: none. Lamia Dahdah: none. Rocco Valluzzi: none. Sara Urbani: none. Carmen Mazzuca: none. Stefania Arasi: Novartis, DBV, Aimmune. Arianna Cafarotti: none. Carla Riccardi: none. Maria Cristina Artesani: none. Lorenza Putignani: none. Valentina Pecora: none. Valeria Marzano: none. Vincenzo Fierro: none.

## Supporting information

Table S1

Table S2

Table S3

Table S4

## Data Availability

Source data available upon request.
