# Supplementary figures and images for: Reactivity to allergenic food contaminants: A study on products on the market
Source: Clin Transl Allergy. 2023 Sep 22;13(9):e12301. doi: 10.1002/clt2.12301 (PMC10515704; doi:10.1002/clt2.12301)

**Supplementary table I** - Guidelines for clinical determination of pass/fail for food challenge


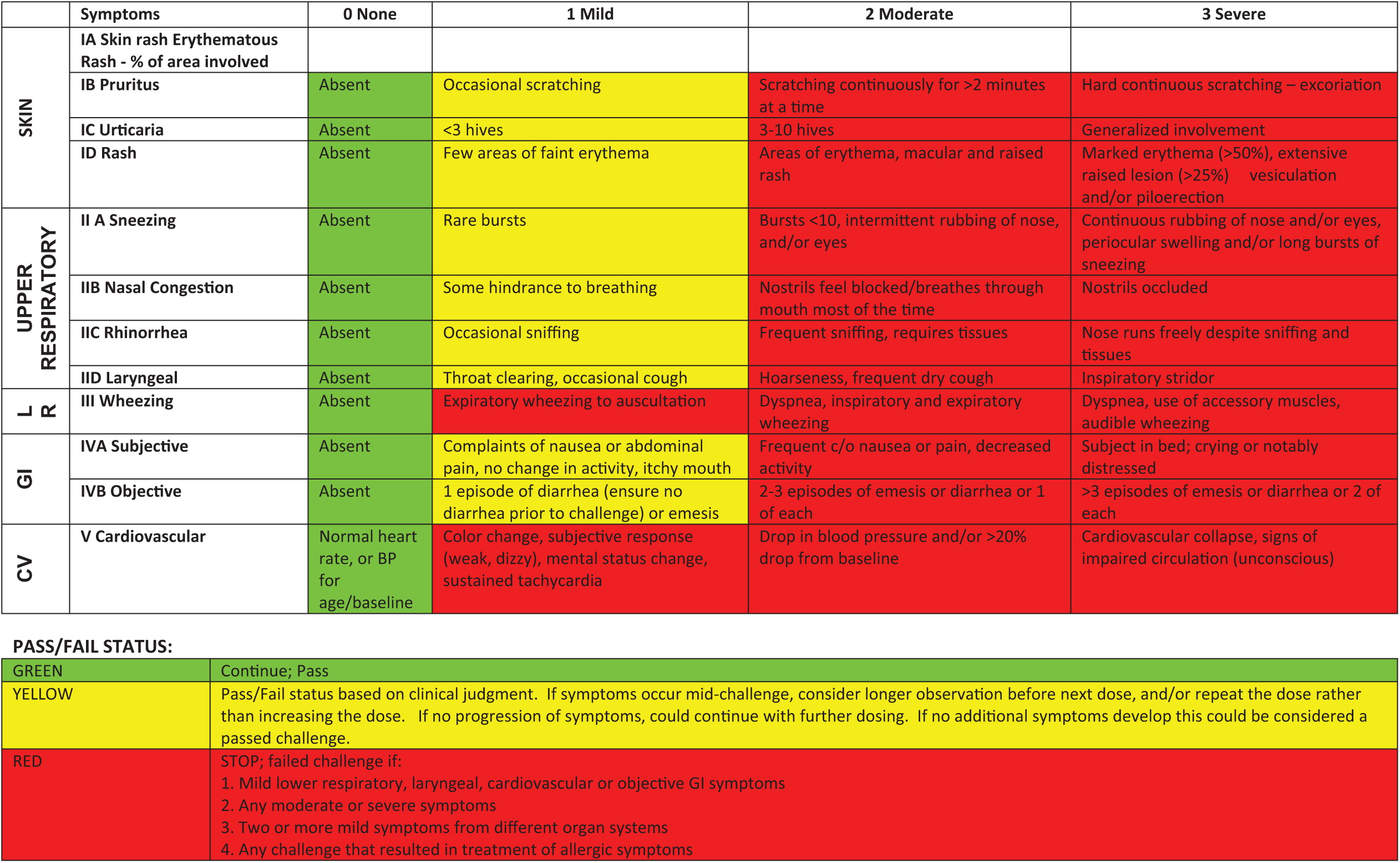

Supplement: Supplementary file 1 — Table S1 [file CLT2-13-e12301-s002.docx]
