# Supplementary material for: Reactivity to allergenic food contaminants: A study on products on the market
Source: Clin Transl Allergy. 2023 Sep 22;13(9):e12301. doi: 10.1002/clt2.12301 (PMC10515704; doi:10.1002/clt2.12301)
Supplement: Supplementary file 2 — Table S2 [file CLT2-13-e12301-s004.docx]

**Supplementary table II.** Group based on grading of symptoms developed during oral food challenge (OFC)

|  |  | | | | |
| --- | --- | --- | --- | --- | --- |
| **Group** | **1** | **2** | **3** | **4** | **5** |
| **Skin** | Localized pruritus, flushing, urticaria, angioedema | Generalized pruritus, flushing, urticaria, angioedema | Any of the previous | Any of the previous | Any of the previous |
| **GI tract** | Oral pruritus, oral “tingling”, mild lip swelling | Any of the previous, nausea and/or emesis | Any of the previous plus repetitive vomiting | Any of the previous plus diarrhea | Any of the previous, plus loss of bowel control |
| **Respiratory tract** | - | Nasal congestion and/or sneezing | Rhinorrhea, marked congestion, **sensation of throat pruritus or tightness** | Any of the previous, **hoarseness, “barky” cough, difficulty swallowing, dyspnea, wheezing, cyanosis** | Any of the previous, **respiratory arrest** |
| **Cardiovascular** | - | - | Tachycardia | Any of the previous, **dysrhythmia and/or mild hypotension** | **Severe bradycardia and/or hypotension or cardiac arrest** |
| **Neurological** | - | Change in activity | Change in activity level plus anxiety | “Light headedness”, feeling of “pending doom” | **Loss of consciousness** |

Boldface symptoms are indications for the use of epinephrine.
