# Supplementary material for: Reactivity to allergenic food contaminants: A study on products on the market
Source: Clin Transl Allergy. 2023 Sep 22;13(9):e12301. doi: 10.1002/clt2.12301 (PMC10515704; doi:10.1002/clt2.12301)
Supplement: Supplementary file 3 — Table S3 [file CLT2-13-e12301-s003.docx]

**Supplementary table III - Selected marker peptides, sequences and precursors for LC-MS/MS milk allergen analysis.**

|  |  |  |  |  |
| --- | --- | --- | --- | --- |
| **Allergenic ingredient** | **Sequence** | **m/z precursor** | **Transition** | **m/z fragment** |
| Milk | FFVAPFPEVFGK | 692,9 ++ | y8  y9  y6  y7 | 920,5  991,5  676,4  823,4 |
|  | NAVPITPTLNR | 598,3 ++ | y8  b3  y6  y5 | 911,5  285,2  701,4  600,3 |
