# Supplementary material for: Reactivity to allergenic food contaminants: A study on products on the market
Source: Clin Transl Allergy. 2023 Sep 22;13(9):e12301. doi: 10.1002/clt2.12301 (PMC10515704; doi:10.1002/clt2.12301)
Supplement: Supplementary file 4 — Table S4 [file CLT2-13-e12301-s001.docx]

**Supplementary table IV - Selected marker peptides (labelled and unlabelled form), sequences and precursors for LC-MS/MS hazelnut allergen analysis.**

| ***Unlabelled peptides*** |  |  |  |
| --- | --- | --- | --- |
| **Sequence** | **m/z precursor** | **Transition** | **m/z fragment** |
| ADIYTEQVGR | 576,4++ | [y7] | 852,3 |
|  |  | [y6] | 689,3 |
|  |  | [y5] | 588,3 |
|  |  | [b6] | 693,2 |
| ALPDDVLANAFQISR | 815,4++ | [y8] | 906,4 |
|  |  | [y7] | 835,3 |
|  |  | [y9] | 1019,6 |
|  |  | [y13] | 1445,7 |
|  |  |  |  |
| ***Labelled peptides*** |  |  |  |
| **Sequence** | **m/z precursor** | **Transition** | **m/z fragment** |
| ADIYTEQVGR* | 581,3++ | [y7] | 862,4 |
|  |  | [y6] | 699,4 |
|  |  | [y5] | 598,3 |
|  |  | [b6] | 693,3 |
| ALPDDVLANAFQISR* | 820,4++ | [y8] | 916,5 |
|  |  | [y7] | 845,5 |
|  |  | [y9] | 1029,6 |
|  |  | [y13] | 1455,7 |
